# Self-Fulfilling Prophecies as a Link between Men’s Facial Width-to-Height Ratio and Behavior

**DOI:** 10.1371/journal.pone.0072259

**Published:** 2013-08-28

**Authors:** Michael P. Haselhuhn, Elaine M. Wong, Margaret E. Ormiston

**Affiliations:** 1 School of Business Administration, University of California, Riverside, Riverside, California, United States of America; 2 Organisational Behaviour Area, London Business School, London, United Kingdom; University of Goettingen, Germany

## Abstract

The facial width-to-height ratio (fWHR) has been identified as a reliable predictor of men’s behavior, with researchers focusing on evolutionary selection pressures as the underlying mechanism explaining these relationships. In this paper, we complement this approach and examine the extent to which *social processes* also determine the extent to which men’s fWHR serves as a behavioral cue. Specifically, we propose that observers’ treatment of target men based on the targets’ fWHR subsequently affects behavior, leading the targets to behave in ways that are consistent with the observers’ expectations (i.e., a self-fulfilling prophecy). Results from four studies demonstrate that individuals behave more selfishly when interacting with men with greater fWHRs, and this selfish behavior, in turn, elicits selfish behavior in others.

## Introduction

Recent research has established men’s facial width-to-height ratio (fWHR) as a remarkably robust predictor of a wide range of behaviors. For instance, men with greater fWHRs are more aggressive [1], less trustworthy [2], and more prone to engaging in deception [3] compared to men with smaller fWHRs. Other research has found positive correlates of men’s fWHR as well–firms led by male CEOs with greater fWHRs achieve superior financial performance [4], and men with greater fWHRs contribute more to group efforts when intergroup competition is made salient [5].

Researchers have generally pointed to evolutionary selection pressures as the underlying mechanisms explaining these relationships. Although early work posited that intersexual selection mechanisms may have formed the links between fWHR and behaviors [1], [4], [6], more recent research has supported an intrasexual selection perspective [7], with growing evidence suggesting that men’s facial structure is an important cue to their ability to obtain resources from others. For example, greater fWHR is associated with baseline testosterone levels [8] (see also [9]) and researchers have argued that exposure to relatively high levels of testosterone may explain the link between greater fWHR and aggressive behavior in men [8], [10]. Thus, it is possible that men with greater fWHRs are biologically predisposed to evolutionarily-beneficial aggressive behavior.

In this paper, we consider a complementary perspective to the evolutionary underpinnings of the relationships between fWHR and behavior. Specifically, we examine the possibility that the link between men’s fWHR and behaviors may also be *socially* driven, as opposed to exclusively *biologically* driven.

In general, seemingly irrelevant physical characteristics may become linked with behaviors as part of a complex interplay between observer perceptions of the trait, observer behaviors as a function of these perceptions, and finally the target individual’s own behaviors in response to how he or she has been treated (e.g., [11]–[13]). In many cases, observers’ initial perceptions shape their behavior in such a way that later elicits the previously anticipated characteristics from the target individual (i.e., a self-fulfilling prophecy). For example, Zebrowitz, Voinescu and Collins [14] found that perceptions of men’s honesty based on childhood facial photographs were associated with actual honesty in adulthood. These results were attributed to the self-fulfilling prophecy effect, such that honest appearances led to greater trust from observers, which subsequently elicited honest behavior.

In the current context, a self-fulfilling prophecy mechanism suggests that observers may treat men with greater fWHRs in ways that elicit the aggressive, self-interested behavior often associated with this trait. Indeed, observers view men with greater fWHRs as more aggressive [15] and less trustworthy [2], [16]. If observers act on these perceptions by preemptively confronting or competing against these individuals (e.g., [17]–[19]), this may lead men with greater fWHRs to respond in kind, thus fulfilling observers’ initial expectations. Such social processes may have long-term effects as well–high versus low fWHR men may be socialized over the course of their lives to show particular patterns of competition, prompted by the differential responses of others (see [20]–[22]).

The purpose of the current research is to provide an initial test of the self-fulfilling prophecy explanation for the link between men’s fWHR and behavior. We first establish a relationship between fWHR and general self-interest, demonstrating that men with higher fWHRs (i.e., our target individuals) behave more selfishly when dividing resources between themselves and a partner. In two subsequent studies, we examine the same resource allocation decisions from the partner’s point of view and show that partners change their own behavior based on a target’s fWHR. In a fourth study, we close the circle by showing that partners’ behavior based on targets’ fWHR leads the target to act in ways that are consistent with partners’ expectations. In this way, we illustrate that a link between men’s fWHR and behavior, which may otherwise be attributed to stable biological factors, is also a function of social responses to men’s facial structure.

### Ethics Statement

Approvals for the studies reported here were obtained from the appropriate human research review committees at London Business School (Study 1) and University of California, Riverside (Studies 2–4). Informed consent information was provided to participants in written form; participants indicated consent by their continued participation in the computer-based studies reported below. In accordance with standard procedures within the psychological sciences, materials and data described here are freely available from the authors upon request.

## Study 1

We examined the relationship between fWHR and behavior in the context of resource allocation decisions using an instrument developed by Van Lange et al. [22]. We chose this particular instrument for two reasons. First, the measure is designed to unambiguously distinguish between self-interested and cooperative preferences for divisions of resources. Although previous research has suggested that men with greater fWHRs are more self-interested (e.g., by keeping money in a trust game or by cheating to increase financial gain [2], [3]), this work has confounded self-interest with other considerations, such as willingness to engage in unethical or untrustworthy behaviors. The second reason for using this instrument is because it has been extensively used as a measure of stable individual differences in social value orientations, or preferences for how resources should be divided between one’s self and others [23]. If self-fulfilling prophecy mechanisms are shown to affect behavior within this context, it would provide evidence for the powerful effect of social factors on what is typically considered to be a dispositional characteristic [22].

The purpose of Study 1 was to establish a general relationship between men’s fWHR and self-interested behavior. Based on the previous research described above, we expected men with greater fWHRs to be primarily concerned about their own outcomes in resource allocation decisions and to show less of a concern for the outcomes of their partners.

### Method

#### Participants

We recruited 131 men from a large European business school. Participants were paid £10.00 for their participation. We did not collect information regarding participants’ age; individuals were drawn from a population ranging from 18 to 69 years of age with an average age of 26 years old.

#### Procedure

Participants completed a resource allocation task as part of a larger set of surveys. After completing the surveys, participants’ photographs were taken for the fWHR measurements.

#### fWHR

Two trained research assistants measured the width and height of each face using NIH ImageJ software. Inter-rater agreement was high for overall fWHR (α = .96).

#### Resource allocations

Researchers have identified three important general preferences (or orientations) for how resources should be divided: prosocial, individualistic and competitive orientations. Prosocial orientations are characterized by a concern for both one’s own outcomes as well as the outcomes of other involved parties. Individualistic orientations are characterized by a concern for one’s own outcomes but little concern for what others receive. Competitive orientations are characterized by a concern for maximizing one’s own outcomes *relative* to those of others (i.e., a desire to outperform others). Although individualists and competitors have different underlying motives for their behavior, in many contexts (e.g., zero-sum games) their specific goals are the same. Thus, researchers often compare prosocial orientations to the combined preference for individualistic or competitive outcomes (*proself* orientations) [24], [25]. We adopt this approach in our research.

To measure participants’ preferences, we employed a measure reported in Van Lange et al. [22]. The measure uses a series of nine decomposed economic games in order to distinguish among the three orientations. Participants are asked to imagine that they will be making choices that will affect both them and an anonymous other person (and that their counterpart will be simultaneously making the same choices for themselves). Each game consists of three possible allocations of points, with the instruction that points should be considered of value. One allocation in each set maximizes the overall points that would be awarded to the decision maker and his counterpart (prosocial option). A second allocation maximizes the points that the decision maker himself will earn (individualistic option). The final allocation maximizes the difference between the decision maker’s points and those of his counterpart (competitive option). The total number of prosocial and proself choices constitutes our dependent measure.

### Results and Discussion

We predicted that men’s fWHR would positively relate to selfish behavior and negatively relate to prosocial behavior in resource allocation decisions. Consistent with our hypothesis, fWHR was a significant negative predictor of the number of prosocial options chosen, *b* = −5.15, *SE* = 2.45, β = −.18, *t*(129) = −2.11, *p* = .037; Model F statistic: F (1, 129) = 4.43, *p* = .037. No control variables were included in this analysis. As prosocial and proself preferences are mutually exclusive, this correlation also indicates that men with greater fWHRs chose significantly more proself options.

Although previous research has focused on differences between prosocial and proself preferences [24], [25], we were able to analyze participants’ decisions for the two sub-dimensions of proself behavior (individualistic and competitive) as well. Breaking down the two dimensions of selfish preferences, facial ratios were marginally positively related to individualistic choices (*b* = 3.90, *SE* = 2.12, β = .16, *t*(129) = 1.85, *p* = .067). No other effects were significant.

The results of Study 1 provide support for our hypothesis that men’s fWHRs predict general orientations toward selfishness versus concern for others. Specifically, men with greater facial ratios were less likely to be characterized by prosocial preferences, and more likely to choose allocations that maximized their own self-interest. Indeed, supplementary analyses suggested that men with greater fWHRs sought to secure as many resources as possible for themselves as opposed to competitively maximizing the difference between their own allocation and that of their counterpart. Although these latter results were only marginally significant and should therefore be interpreted with caution, they may provide some insight into past research that has confounded exclusive self-interest with actions that benefit one’s self while actively harming another party [2], [3]. Perhaps in the absence of direct provocation, men with greater fWHRs are primarily concerned for their own well-being and are less concerned with the well-being of others in either a positive or negative direction. Overall, these results of the current study are consistent with the behavioral correlates of fWHR demonstrated in previous research, and provide evidence that a target individual’s fWHR is a predictor of his general approach to resolving conflicts over scarce resources.

Beyond these implications, the instrument used in this study has frequently been considered a measure of dispositional differences in selfish versus cooperative orientations (e.g., [24], [26]). Thus, it could be argued that the results of Study 1 provide evidence of a link between men’s facial structure and stable, individual differences in self-interest. In the next study, however, we begin to examine the possible *social* underpinnings of this relationship by studying whether observers shape their behavior in these economic games based on the target’s fWHR.

## Study 2

Individuals’ emotions and behavior in social interactions are often based on their expectations for how their counterpart may behave. For example, individuals respond positively to a counterpart’s apparent distress in competitive contexts [27] and act more competitively in negotiations when they anticipate competitive behavior from a counterpart [19]. As previously noted, men with greater fWHRs are perceived to be more aggressive and less trustworthy. Thus, we predicted that individuals will act more selfishly (and less cooperatively) when they believe that they are interacting with a man with a greater fWHR compared to a man with a smaller fWHR.

### Method

#### Participants

We recruited 173 U.S. participants through Amazon Mechanical Turk. Individuals were paid $.50 for their participation. Thirteen participants failed to complete the study and were dropped from further analyses.

To ensure the quality of the data collected from this sample (i.e., online, anonymous participants), five research assistants were asked to complete the task as quickly as possible while maintaining the accuracy and quality of their responses. The fastest completion time recorded was approximately two minutes. We therefore conservatively eliminated participants who finished in half of the fastest time (i.e., under one minute). This resulted in the removal of two individuals from the remaining analyses bringing our final sample to 158 participants (46% male, Age: *M* = 31.49, *sd* = 12.67); the pattern of results remains the same if these individuals are included.

#### Procedure

Participants completed the resource allocation task described in Study 1. In this study, participants were shown the face of their anonymous counterpart. As in Study 1, it was explained that participants’ decisions would affect both them and their counterpart, and that their counterpart would simultaneously be making their own choices for each economic game. Approximately half of the participants were randomly assigned to a *high-fWHR counterpart* condition (*n* = 82) in which their ostensible counterpart had a relatively large fWHR, and the remainder of the participants to a *low-fWHR counterpart* condition (*n* = 76) in which their ostensible counterpart had a relatively small fWHR. In each of the nine economic games, participants were shown a photograph of their counterpart, asked to imagine how their counterpart might behave in this game, and then to make their own decision as to their preferred allocation.

#### fWHR manipulation

We obtained our photographs from a database created by the Karolinska Institute [28] that has been used in previous studies of facial perception [29]. The database contains facial photographs of 33 Caucasian males with neutral facial expressions. The first author and two research assistants measured the fWHRs of these men using the procedures detailed in Study 1 (α = .96). We selected the men with the four greatest (*M* = 1.90, *sd* = .02) and four smallest (*M* = 1.54, *sd* = .03) fWHRs to use in our study. We elected to use four examples of both high and low fWHR to smooth over any idiosyncrasies in the individual photographs.

#### Resource allocations

Participants completed the same resource allocation task from Study 1.

### Results and Discussion

#### Preliminary analyses

No significant differences in prosocial or proself choices emerged between the participants exposed to the specific faces used in either the high-fWHR (*F*(3,78) = 1.22, *p* = .31) or low-fWHR (*F*(3,72) = .67, *p* = .57) counterpart conditions. Therefore, we collapsed the data across photographs within each experimental condition.

#### Resource allocations

We predicted that individuals paired with a high-fWHR counterpart would act more selfishly compared to individuals paired with a low-fWHR counterpart. Consistent with this prediction, participants in the high-fWHR counterpart condition selected significantly fewer prosocial options across the nine economic games (*M*s = 4.41 vs. 5.86 *sd*s = 3.79 and 3.89), *F*(1,156) = 5.55, *p* = .02. We observed no significant main effect or interaction with participants’ gender.

We once again conducted supplementary analyses to examine the two sub-dimensions of proself allocations. Participants in the high-fWHR counterpart condition selected marginally more individualistic options compared to those in the low-fWHR counterpart condition (*M*s = 3.49 vs. 2.51, *sd*s = 3.56 and 3.58), *F*(1,156) = 2.94, *p* = .09. No other effects were significant.

The results of Study 2 highlight the power of men’s facial structure in shaping observers’ behavior in resource allocation decisions. Participants who imagined that they were dividing resources with a relatively high-fWHR counterpart behaved significantly more selfishly compared to those who imagined that they were interacting with a relatively low-fWHR counterpart. These results are consistent with previous work demonstrating that observers are generally wary when interacting with high-fWHR men (e.g., [2]).

Intriguingly, our supplementary analyses suggested that individuals who imagined that they were allocating resources between themselves and a relatively high-fWHR man may make individualistic choices (i.e., choices that maximized their own outcomes) as opposed to competitive choices (i.e., choices that maximized the difference between their outcomes and those of their high-fWHR counterpart). Although we once again emphasize that caution should be taken in interpreting marginally significant results, these findings mirror those of Study 1 in which greater fWHRs were associated with more individualistic, as opposed to competitive, behavior in men.

Although these results provide initial support for our prediction that high-fWHR men will be treated differently than low-fWHR men, there are some limitations to the current study. First, the stimulus materials for the study were composed of photographs of different individuals. Although using such materials enhances the external validity of these results, it also introduces the possibility that idiosyncratic differences between individuals (e.g., hair length, skin color) may have affected these results. A second limitation of the current study is that it does not directly measure the processes underlying the treatment of high-fWHR versus low-fWHR men. To address these potential concerns, we conducted a follow-up study to more conclusively establish the relationship between men’s fWHR and counterpart behavior.

## Study 3

Study 3 was designed both to replicate the findings of Study 2 as well as to build on the previous study by addressing its limitations. Specifically, in Study 3 we employed computer-manipulated photographs of the same individual to test whether variations in fWHR, independent of other factors, affected counterpart behavior. In addition, we tested our prediction that counterpart perceptions would underlie the behavioral differences demonstrated in Study 2 by asking participants to predict how their partner would behave in the resource allocation task used in Studies 1 and 2. We expected that individuals “paired” with a high-fWHR individual would anticipate more selfish behavior from their ostensible counterpart (compared to those “paired” with a low-fWHR individual), and these expectations would directly result in less prosocial behavior.

### Method

#### Participants

We recruited 255 U.S. participants through Amazon Mechanical Turk. Individuals were paid $.50 for their participation. Twelve participants failed to complete the study and were dropped from further analyses.

Once again, to ensure the quality of the data, three research assistants were asked to complete the task as quickly as possible while maintaining the accuracy and quality of their responses. The fastest completion time recorded was approximately eight minutes. We therefore conservatively eliminated participants who finished in half of this time (i.e., under four minutes). This resulted in the removal of 36 individuals from the remaining analyses bringing our final sample to 207 participants (57% male, Age: *M* = 32.01, *sd* = 12.40); the pattern of results remains the same if these individuals are included.

#### Procedure

The materials were included in a set of other, unrelated studies. The task and procedures were identical to those of Study 2 with two exceptions. First, the fWHR manipulation employed different stimulus materials. Second, prior to making their own resource allocation decisions, participants predicted which option their counterpart would choose for each of the nine allocation decisions.

#### fWHR manipulation

We obtained photographs from materials developed by Stirrat and Perrett [2]. These materials include manipulated photographs of 17 Caucasian men. Specifically, the facial structure of each man was manipulated in order to create a version of the same individual with high-fWHR and low-fWHR. Three manipulation procedures were used to ensure that any perception differences based on fWHR were due to men’s facial structure, rather than any artifacts of the specific manipulation process (see [2] for details). Stirrat and Perrett reported no significant effects of the specific manipulation procedure, nor of the specific individuals used in the materials. Thus, we randomly selected two individuals and their corresponding high-fWHR and low-fWHR photographs (i.e., four total photographs) from one of the manipulation procedures to use in our study.

#### Resource allocations

Participants completed the same resource allocation task from Study 1 in two steps. First, participants were shown a photograph of their ostensible counterpart and were asked to predict the decision that he would make for each game. Second, participants made their own decisions for each game.

### Results and Discussion

#### Preliminary analyses

We first tested for differences between the two different men used in the stimulus materials. Marginally significant differences emerged for expectations of counterpart behavior in the high-fWHR condition (*F*(1,103) = 2.73, *p = *.10) and for one’s own prosocial choices in the low-fWHR condition, *F*(1,100) = 2.19, *p = *.10. No other effects were significant. Due to the marginally significant differences, we conducted our primary analyses both with and without controlling for the specific face viewed by the participant. The pattern and significance of our results were identical; we report the results of the analyses without the control variable below.

#### Expectations of counterparts’ resource allocations

We predicted that individuals paired with a high-fWHR counterpart would anticipate more selfish behavior compared to individuals paired with a low-fWHR counterpart. Consistent with this prediction, participants in the high-fWHR counterpart condition anticipated significantly fewer prosocial options across the nine economic games (*M*s = 2.99 vs. 4.48, *sd*s = 3.52 and 3.65), *F*(1,205) = 8.94, *p* = .003. We observed no significant main effect or interaction with participants’ gender.

Supplementary analyses revealed that participants in the high-fWHR counterpart condition anticipated significantly more individualistic options compared to those in the low-fWHR counterpart condition (*M*s = 4.61 vs. 3.48, *sd*s = 3.64 and 3.31), *F*(1,205) = 5.44, *p* = .021. No other effects were significant.

#### Resource allocations

We expected that individuals’ expectations of their counterparts’ behavior would subsequently shape their own decisions of whether to demonstrate prosocial behavior. Consistent with this prediction, participants in the high-fWHR counterpart condition selected significantly fewer prosocial options compared to those in the low-fWHR counterpart condition (*M*s = 4.30 vs. 5.36, *sd*s = 3.90 and 3.77), *F*(1,205) = 4.01, *p* = .047. We observed no significant main effect or interaction with participants’ gender.

Supplementary analyses revealed that participants in the high-fWHR counterpart condition selected significantly more individualistic options compared to those in the low-fWHR counterpart condition (*M*s = 3.64 vs. 2.59, *sd*s = 3.75 and 3.27), *F*(1,205) = 4.60, *p* = .033. No other effects were significant.

We expected that the effect of counterpart fWHR on prosocial behavior would be mediated by expectations of counterparts’ behavior. To test this prediction, we conducted a bias-corrected bootstrapping analysis with 5,000 resamples [30] to test the indirect effect of counterpart fWHR on prosocial behavior with anticipated counterpart behavior as a mediating variable. This analysis revealed a significant indirect effect of counterpart fWHR, Mediated effect = .85, *SE* = .30, 95% *CI* = .27–1.47. As the confidence interval does not bridge zero, this analysis supports our hypothesis that anticipated counterpart behavior mediates the relationship between counterpart fWHR and resource allocation decisions.

The results of Study 3 once again demonstrate that men’s facial structure is an important social cue that affects not only observers’ perceptions, but also their behavior. Using manipulated photographs that control for possible confounds, we found that participants who imagined that they were dividing resources with a relatively high-fWHR counterpart expected selfish behavior from their partner and responded in kind. Conversely, participants who believed that they were interacting with a relatively low-fWHR counterpart anticipated more cooperative behavior from their partner and responded by behaving more cooperatively themselves. Consistent with the marginally significant results from the previous studies, individuals in the current study anticipated more individualistic (as opposed to competitive) behavior from high-fWHR counterparts and responded in kind.

## Study 4

Study 1 demonstrated that men’s fWHR predicts how they behave in economic games, and Studies 2 and 3 illustrated how men’s fWHR shapes partners’ behavior. The purpose of Study 4 was to examine how these processes may be linked. Specifically, we predicted that observers’ treatment of target men based on their fWHR would subsequently affect these targets’ behavior, leading them to behave in ways that are consistent with the observers’ expectations. To test this prediction, we exposed participants to the distinct behaviors elicited by the high-fWHR and low-fWHR faces in Study 2 (i.e., greater selfish behavior by those interacting with a high-fWHR counterpart). We examined whether participants’ behavior varied depending on whether they were treated *as if* they were a man with relatively high or low fWHR. Our expectation was that individuals who were treated as if they are a high-fWHR man would respond by behaving relatively selfishly whereas individuals who were treated as if they are a low-fWHR man would respond by behaving relatively prosocially.

### Method

#### Participants

We recruited 218 U.S. participants through Amazon Mechanical Turk. Participants were paid $.50 for their participation. Seventeen participants failed to complete the study and were dropped from further analyses.

The general study design was identical to that of Study 2. To ensure the quality of the data we followed our conservative procedure of eliminating participants who completed the study in less than one minute. This resulted in the removal of one individual from the remaining analyses bringing our final sample to 200 participants (59% male, Age: *M* = 32.43, *sd* = 11.54); the pattern of results remains the same if this individual is included.

#### Procedure

Participants completed the same resource allocation task described in Study 1. In a departure from the previous studies, participants were informed of their counterpart’s decision in each game prior to making their own selection. Participants were randomly assigned to either a high-fWHR treatment condition in which they were treated as if they were a high-fWHR man (*n* = 101) or a low-fWHR treatment condition in which they were treated as if they were a low-fWHR man (*n* = 99). Thus, participants faced different “counterpart” behavior as a function of their experimental condition.

#### fWHR treatment manipulation

Prior to making their own decisions in the resource allocation task, participants were informed of the decision that their ostensible counterpart had made for each of the nine decomposed games. The two conditions were based on actual decisions made in Study 2. Participants in the *high-fWHR treatment* condition were presented with the modal choices made for each of the nine decomposed games in the high-fWHR condition in Study 2. These choices included three “prosocial” selections (games 2, 5 and 7) and six “proself” selections (operationalized as individualistic choices; games 1, 3, 4, 6, 8 and 9). Participants in the *low-fWHR treatment* condition were presented with the modal choices made for each of the nine games in the low-fWHR condition in Study 2. The “prosocial” option was the modal selection in each of the nine rounds.

#### Resource allocations

Participants completed the same resource allocation task from the previous studies.

### Results and Discussion

Preliminary analyses revealed a main effect of gender such that women selected significantly more prosocial allocations (and thus significantly fewer proself allocations) than did men, *M*s = 6.11 vs. 4.32, *sd*s = 3.56 and 3.86, *F*(1,198) = 11.15, *p* = .001. Assessing the separate proself dimensions showed that women made significantly fewer individualistic choices than did men, *M*s = 2.42 vs. 4.16, *sd*s = 3.32 and 3.81, *F*(1,198) = 11.26, *p* = .001. No other effects were significant. Gender did not interact with the fWHR treatment conditions, nor did the pattern of results of our primary analyses change when gender was included as a control variable. Thus, the analyses below did not include gender as a covariate.

We predicted that individuals who are treated as though they are high-fWHR men will respond with less cooperative behavior compared to individuals who are treated as though they are low-fWHR men. Consistent with this prediction, participants in the high-fWHR treatment condition selected significantly fewer prosocial options across the nine games compared to participants in the low-fWHR treatment condition (*M*s = 4.25 vs. 5.89, *sd*s = 3.64 and 3.87), *F*(1,198) = 9.56, *p* = .002. These results illustrate that simply treating people as if they are men with relatively high fWHR yields more selfish behavior.

Supplementary analyses revealed that participants in the high-fWHR treatment condition selected significantly more individualistic options (*M*s = 4.14 vs. 2.73, *sd*s = 3.63 and 3.67), *F*(1,198) = 7.49, *p* = .007. No other effects were significant. These results mirror those of Study 1 in which greater fWHR was associated with more individualistic behavior. Moreover, this study suggests that partners’ behavior based on targets’ fWHR may lead targets to act in ways that are consistent with partners’ expectations. In this way, these findings provide initial evidence that a link between men’s fWHR and behavior may lie in social responses to men’s facial structure.

## General Discussion

Across four studies, our results illustrated a self-fulfilling prophecy explanation for the link between men’s fWHR and behavior. Although men with greater fWHRs behaved more selfishly in what is considered to be a measure of dispositional value orientations (Study 1), further examination suggested that social processes may play a substantial role as well. Specifically, people showed more caution when interacting with a high-fWHR male by protecting their own resources (Studies 2 and 3). We then demonstrated that these observer expectations elicited selfish patterns of behavior (Study 4). In this way, observer perceptions of men based on their fWHR, in addition to any underlying biological characteristics associated with this facial trait, may explain the previously established robust correlations between fWHR and behavior.

Although our study focused on immediate reactions to exposure to selfish behavior, other research suggests that repeated exposure to such behavior from others may shape high-fWHR men’s general dispositions as well. For example, Van Lange et al. [22] demonstrated that patterns of social interaction shape individuals’ social value orientations, such that repeated exposure to situations that require cooperation, or to generally cooperative individuals, encourages the development of a prosocial orientation. In contrast, individuals exposed to non-cooperative, self-interested behavior by others are more likely to develop a proself orientation. Our results suggest that men with greater fWHRs experience less cooperation and more competition from others compared to men with smaller fWHRs, and these differences in exposure to social interactions may also affect men’s general predisposition to cooperate or compete (see Study 1).

The current research illustrates the power of social perceptions in shaping individuals’ behavior. However, it is important to revisit the possibility that biological and social factors may work in concert to strengthen the links between fWHR and behavior. For example, higher levels of testosterone observed in men with greater fWHRs [8] may predispose them to more aggressive behavior, and these natural tendencies are then amplified through social interactions with others as observers are exposed to these actions. Similarly, to the extent that greater fWHR is associated with perceptions of masculinity, expectations regarding men’s physical dominance may also play a role in observers’ initial beliefs and behavior toward these individuals. Another possibility is that although men’s fWHR is, on its own, a seemingly irrelevant cue in social contexts, perhaps this characteristic is correlated with other physical factors that have (or had) greater importance in social interaction. For instance, observers are able to reliably predict men’s physical strength based on facial photographs [31], due in part to perceived links between facial masculinity and physical prowess [32]. Future research should expand upon these possibilities to better understand observers’ initial inclinations to behave more cautiously toward men with greater fWHRs.

A final point of consideration is the fact that, with the exception of Study 1, we did not account for participants’ fWHRs. This is a potentially important omission as male participants’ fWHRs may have influenced their decisions in the social value orientation instrument. On the one hand, not accounting for these potential effects introduces noise in our studies, making for a more conservative test of our hypotheses. On the other hand, intriguing interactions between observers’ and actors’ facial structures may have been overlooked. In particular, it is important to examine how *relative* differences in fWHR affect male-male interactions.

Defining fWHR as high or low depends on the standard to which a particular individual is being compared. To the extent that fWHR is a situational marker for male dominance, it may be the case that men behave more aggressively among men who have comparatively smaller fWHRs but more cautiously among men who have comparatively greater fWHRs. Another possibility is that men with greater fWHRs relative to societal averages are socialized to act in a self-interested manner and this predilection persists regardless of situational factors. In contrast, men with smaller fWHRs relative to societal averages may be socialized to generally behave more cautiously regardless of the situational context. Future research should disentangle these differing perspectives and examine the extent to which either or both explanations are valid.

## Conclusions

Recent research has highlighted the importance of men’s fWHR as a social cue and has focused extensively on potential biological and evolutionary theoretical underpinnings of these relationships. The current article illustrates how social processes, in addition to possible biological differences, can elicit different patterns of behavior as a function of men’s facial ratios.
